# Value and limitations of sonography in kidney transplant recipients with special attention to the resistive index – An update

**DOI:** 10.3389/fneph.2022.997839

**Published:** 2022-09-30

**Authors:** Julia Stigler, Martin Tiefenthaler

**Affiliations:** Department of Internal Medicine IV (Nephrology and Hypertension), Medical University Innsbruck, Innsbruck, Austria

**Keywords:** kidney transplantation, post-transplant complications, ultrasound, color coded Duplex sonography, resistive index

## Abstract

Kidney transplantation has become the standard treatment for end-stage renal disease. Even though the success rates are high, early and late post-transplant complications remain a major clinical problem due to the risk of graft failure. Therefore, it is of highest interest to early diagnose post-transplant complications. Ultrasound with color coded Duplex analysis plays a crucial role in imaging mechanical and vascular complications. In this article, we give an update of the visualizable complications in kidney transplant recipients and discuss the value of resistive index (RI) measurement with its limitations in allograft rejection.

## 1 Introduction

Sonography is one of the most important tools in diagnostic monitoring of kidney transplants. It is easily available and can be performed quickly with a lack of radiation, nephrotoxic contrast agent and low continuous costs by the nephrologist. Utilizing a curved arrow probe (4-8MHz), most of the severe pathologies causing graft dysfunction including vascular/mechanical complications, urologic disorders and adverse immunologic effects can be visualized. Perfusion is estimated by color coded Duplex sonography (CCDS, 2,5MHz) with a standardized setting in extension to RI measurements. Contrast-enhanced ultrasound (CEUS) plays an emerging role in the post-kidney transplant setting in focal lesions.

In combination with hydration measurements as the diameter of the inferior Vena cava (in the pars hepatica) and its response to aspiration (“cavography”), sonography gives the first clue in unclear decrease in kidney function and thus is part of the basic nephrology training in many curriculas.

## 2 Indications

Sonography should regularly be performed as a routine ultrasound in the context of clinical follow ups. Especially in all cases of graft dysfunction sonography is indicated to evaluate the morphology and perfusion of the graft for signs of acute tubular necrosis, graft rejection and drug nephrotoxicity and to check for urinary tract abnormalities, external compression by edema or hematomas and for obstruction of vessels by lymphoceles and ureteral strictures (1).

## 3 Routine sonography

### 3.1 B-imaging (high resolution 2D sonography)

First, the size of the kidney and the thickness of the parenchyma (in relation to the pyelon) is measured. The size of the transplant kidney can only be interpreted by intraindividual changes, whereby graft swelling or enlargement are alert signs. The entire graft is scanned for cysts and for changes within the parenchyma: There can be decreased echogenicity, heterogeneous areas of increased or decreased echogenicity, obscured corticomedullary differentiation and not delimitable or prominent mark pyramids. Next, the renal pelvis is checked for hydronephrosis.

Furthermore, the perirenal area is scanned *via* B-imaging to check for any fluid collections (e.g. hematomas, lymphoceles, urinomas…) (1, 2).

### 3.2 Color coded Duplex sonography (CCDS)

With duplex sonography, perfusion density and distribution is measured. With a standard CCDS setting for transplanted kidney gross perfusion loss can be estimated. There are two helpful parameters: POV = percentage of vascularization (normal > 30%-50%), which describes the amount of colored content in relation to the non-colored renal parenchyma. PVD = periphery vessel distance (normal < 0.25 cm) measures the distance between the most outside located visible vessels by standard CCDS and the renal capsule (3).

A reduction in POV can be caused by infiltration/swelling, fibrosis, intravasal hypovolemia, and hypotension, whereas an increase in POV is mostly concomitant with an increase in vascularization (polyoma reactivation, acute tubular necrosis, fever, sepsis).

An increase in PVD is often seen in old to old organ transplantation, in toxic CNI levels, and by mechanical compression. Also iatrogenically, an increase in PVD can be caused by too much pressure with the transducer.

Even though these two parameters have not become the international standard, we still regard them as helpful tools for the description of the overall-vascularization of the transplant.

The resistive index (RI) is measured using spectral Doppler at the arcuate arteries (at the corticomedullary junction) or interlobar arteries (adjacent to medullary pyramids). One should especially check for pulsatile flow, causing an elevated RI.

CCDS can also visualize arteriovenous (AV) fistula, segmental or total loss of perfusion, vessel stenosis and urinary tract stones by twinkling.

### 3.3 Contrast enhanced ultrasonography (CEUS)

CEUS is a fast and safe technique that can complement ultrasound even at patients’ bedside. Briefly 1,2 ml of Sonovue(^®^) are infused while monitoring a suspect lesion with conventional and Contrast Sonography in parallel for a complete cycle of arterial and venous phase. The reflection of Doppler signal on the microbubbles of the contrast agent is specific for different lesions in regard to their perfusion.

CEUS is able to exploit the main vascular, urological, and parenchymal complications, improving the diagnostic performance of grayscale ultrasound and CCDS examination with higher specificity.

It plays an emerging role in the diagnosis of any perfusion problem, since it can exploit hypoperfused or infarcted parenchymal areas.

**Figure 1:**
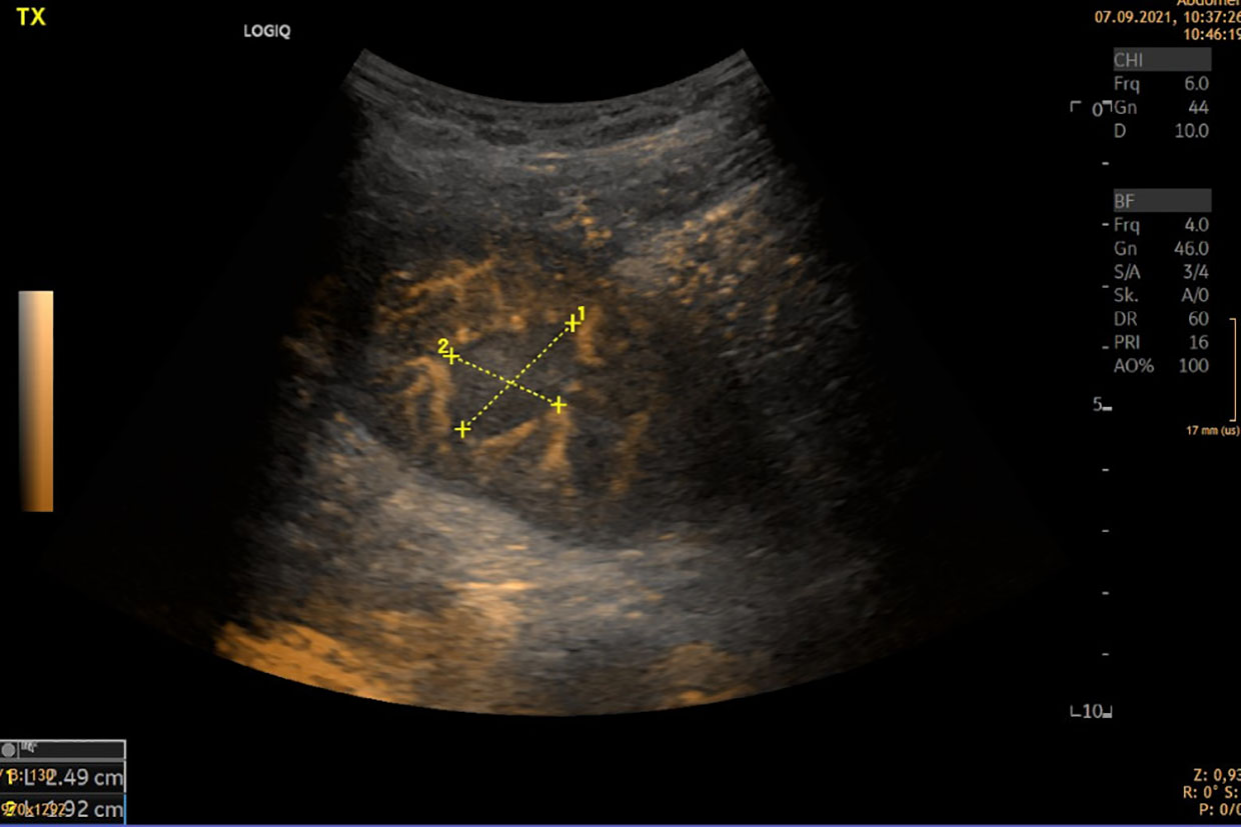
CEUS. Hypoperfused area with PET-CT correlation as pyelonephritis.

Also in acute pyelonephritis, CEUS was shown to have a good sensitivity and specificity (4). We show in **Figure 1** an otherwise undetectable abscess in a hypoperfused area.

The role of CEUS in detecting parenchymal lesions or neoplasms is already well-known from non-transplanted patients and can be transmitted to kidney transplant recipients (5).

To our knowledge to date there is no clear role or additional value of shear wave elastography in the examination of the transplanted kidney.

## 4 Pathological findings and collecting system abnomalities

### 4.1 Perinephric Fluid Collections after Kidney Transplant

#### 4.1.1 Lymphoceles

Lymphoceles may become very large. They can either arise from donor lymphatics (mostly posteromedial to the allograft with a risk to obstruct the proximal ureter) or – less commonly - from native lymphatics *via* disruption during vascular anastomosis with risk to obstruct venous drainage of the leg, leading to ipsilateral edema. The most dreaded complication is a compression of the venous anastomosis (1). Lymphoceles should not be confounded with bladder diverticuli, ascites or ovarian cysts.

Because of frequent relapse after punction, marsupialisation or windowing are the preferable therapeutic options!

### 4.1.2 Urinomas

Perirenal fluid collections can also be urinomas, due to ureteral necrosis, due to dissection of the renal hilus, or due to leakage from the anastomosis with the bladder.

The appearance can be identical to that of a lymphocele, whereby dilatation of the collecting system proximal to the leak is common. The diagnosis should be confirmed by laboratory analysis after fluid aspiration under sonographic guidance.

### 4.1.3 Hematomas

are heterogeneous, containing both liquid (anechoic) and solid (echogenic) components, occuring as a result of surgery or percutaneous biopsy. Particularly subcapsular hematomas, with compression of the remaining parenchyma termed as Page kidney should be detected.

### 4.1.4 Seromas

Seromas are related anechoic fluid collections that are extremely common in the immediate postoperative period.

### 4.1.5 Pyelonephritis

Pyelonephritis can also be an acute cause of allograft failure. The B-imaging signs are diffuse or local cortical enlargement, appearing as localized, poorly marginated hypoechoic regions caused by interstitial edema. Doppler sonography has an improved sensitivity in detecting parenchymal abnormalities, as most pyelonephritic lesions are ischemic. These are better identified by power Doppler US than by CCDS (6). Contrast-enhanced US (CEUS) can improve the detection of low flow (4, 7) (**Figure 1**).

### 4.1.6 Hydronephrosis

Hydronephrosis can be categorized by a grading system (grade I through IV).

In most kidney transplants, ampullary pelvis (with extended calyces) up to hydronephrosis °I (confluence of some calyces, but remaining separation of the calyces) is seen. Hydronephrosis °I may be caused by denervation of the ureter - and thus is “normal” in transplant kidneys - and may be related to back pressure from the bladder. In 2-5% of cases, urinary obstruction with dilated renal pelvis and confluence of all calyces(°II) and loss of separation of pelvic segments (°III) is seen. Other causes can be edema of ureteral anastomosis, infection, compression by perinephric fluid collections (lymphocele, hematoma) or ischemia causing stricture (2, 8). Moreover, BK virus reactivation is associated with ureteral stenosis (9). The site most commonly involved is the anastomosis into the bladder.

**Figure 2:**
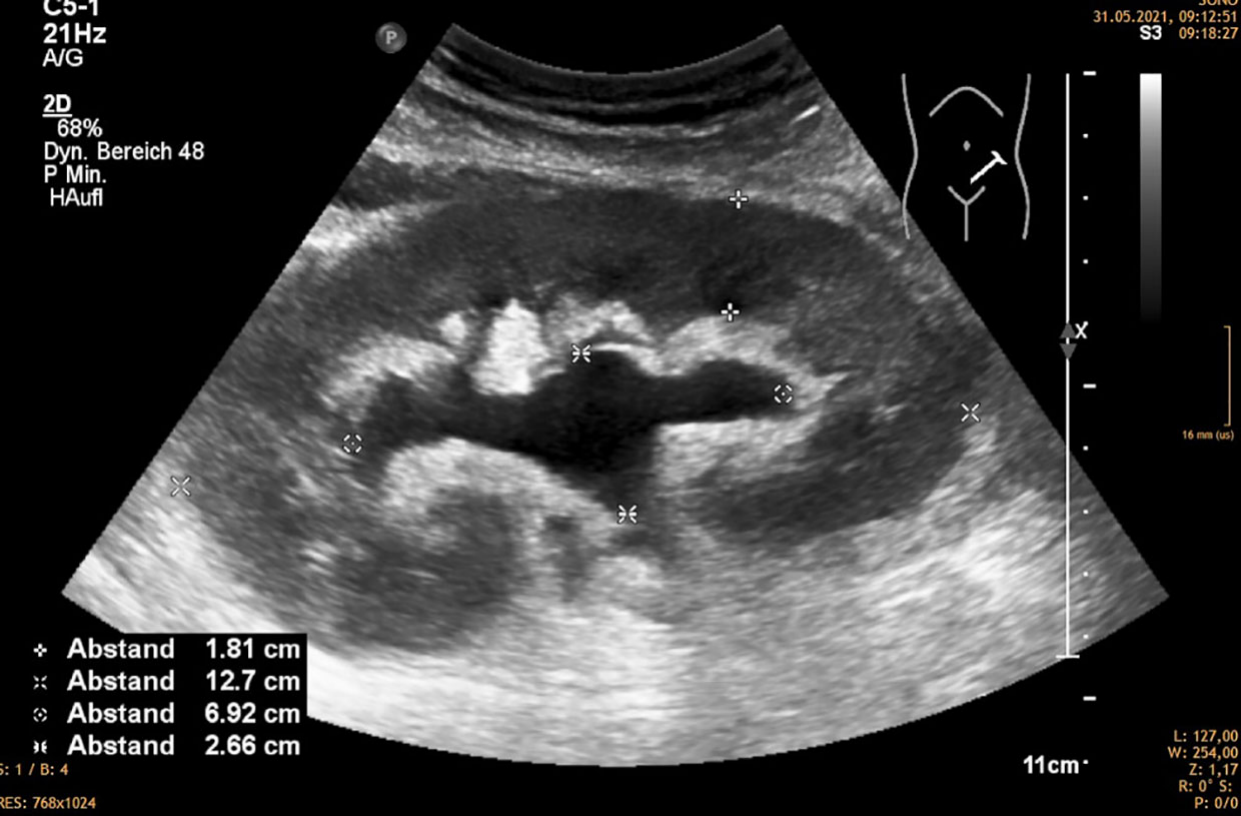
Hydronephrosis 2 without distended ureter, mostly caused by compression of the exterior of the kidney by lympocele, hematoma, etc.

Hydronephrosis without dilatation of the proximal ureter indicates obstruction at the ureteropelvic junction. This is commonly caused by extrinsic compression by a lymphocele (1) (**Figure 2**).

## 5 Vascular complications

In a study by Aktas et al. renal artery stenosis is the most common vascular complication (usually occurring within the first 12 months after transplant, 3-15%), followed by allograft renal artery kinking, renal vein kinking, renal artery thrombosis, renal vein laceration, renal artery laceration, renal vein thrombosis, renal artery disruption and vein obstruction due to pressure from a lymphocele or a hematoma (10).

Many of these complications can be treated by interventional radiologic procedures. Still, the restenosis rate is between 12-28% (11, 12).

### 5.1 Transplant renal artery stenosis (TRAS)

TRAS is usually a late(r) complication, occurring 3-24 months after surgery (13).

Mostly, stenosis is on the anastomosis site itself (iatrogenic). If multiple segments of the donor artery are stenotic, this is usually due to the catheter-related trauma to the intima during the phase of cold ischemia. Moreover, torsion/curving of the renal artery can highly affect hemodynamics. And also an upstream stenosis of the iliac vessels can cause similar phenomena. A too long TRA may cause kinking and the findings of a stenosis. Usually, this complication needs surgical reconstruction.

Risk factors for TRAS include atherosclerotic disease in the donor vessels, cytomegalovirus infection, delayed restoration of renal function, and transplantation of a pediatric kidney in an adult recipient (14).

The presence of reduced organ volume in chronic graft dysfunction and a focal high peak systolic velocity (PSV >180-200 cm/s) is suggestive for significant TRAS. In CCDS stenotic segments appear as focal areas of color aliasing. Acceleration of flow that is 2.5 times higher than the pre- or post-stenotic velocity represents a direct criterion for the diagnosis of TRAS. A PSV of greater than 250cm/sec predicts significant stenosis with high sensitivity and specificity (15, 16).

Other criteria are non-specific, e.g. a very low intrarenal RI (< 0.5) (17) and the tardus-parvus-waveform (a waveform which has a delayed or slow rise to the systolic peak “tardus” and a diminished systolic amplitude “parvus” creating a rounded systolic peak) in the distal renal artery. An acceleration time > 0.06 sec can be specific if TRAS is > 80% (16, 18).

### 5.2 Arteriovenous fistulas (AVFs) and pseudoaneurysms (PAs)

Arteriovenous fistulas occur in up to 10%–16% of renal allograft biopsies (19, 20) and may only be detected with CCDS. We therefore monitor all patients at latest one day after the biopsy (21). AVFs are areas of disorganized flow, where the artery has a low impedance waveform, the vein is arterialized.

However, about 70% of AVFs are asymptomatic and are resorbed spontaneously within weeks (20). Larger or symptomatic lesions accompanied by hematuria, hypovolemia, renal insufficiency, hypertension, and/or high output cardiac failure require interventions such as super‐selective trans‐catheter embolization (22). In rare cases, AVFs are large enough to reduce renal perfusion and cause graft ischemia. Patients with large AVFs, who undergo repeated renal biopsies, are at increased risk for hemorrhagic complications.

Pseudoaneurysms have the typical pattern of high flow during systole and outflow during diastole. They are at risk to rupture, and for this reason, they should always be treated (16).

### 5.3 Renal vein thrombosis (RVT)

RVT is suspected clinically when there is sudden diminished urine output, graft tenderness and enlargement, and elevated serum creatinine as well as proteinuria in laboratory findings (1). Causes of RVT are compression of the renal vein by fluid collections (lymphocele, urinoma etc.), propagation of clot from the iliac vein and hypovolemia. B-mode examination reveals renal enlargement, reduced parenchymal echogenicity, diminished/absent corticomedullary differentiation, and disappearance of the renal sinus and collecting system (all of which are nonspecific). The two most important CCDS findings are the absence of the venous color signal (reflecting absence of vascularization) and reverse diastolic flow within the renal artery (23, 24).

Renal graft venous thrombosis is an early complication, usually occuring during the first 2 weeks post transplantation. The incidence is between 0.3-6.1% (25, 26). The most common predisposing factors are mechanical obstructions (24). Therapy can either be interventional radiologic or by surgical thrombectomy. Still, thrombosis is difficult to treat and if it is complete, it usually leads to graft loss. Therefore it is essential to avoid renal graft venous thrombosis by correct placement of the venous anastomosis (avoiding torquation) and stringent fluid management when polyuria is occuring.

### 5.4 Other early vascular complications

An occlusion involving a segmental artery leads to segmental infarction, which is reflected by the absence of arteriovenous flow only in the affected segment. Therefore, Power Doppler imaging can be helpful to identify the low-flow vessels. CEUS is especially useful to visualize infarction and cortical necrosis.

## 6 Parenchymal disorders leading to graft dysfunction

Delayed graft function occurs in 3-35% of kidney transplant recipients, mainly in deceased donors’ transplants. One major pathophysiological aspect is the ischemia-reperfusion injury. Still, the prediction of delayed graft function is difficult.

It was often proposed that RI elevation is linked to increased rates of allograft rejection and long-term mortality (27, 28). The RI is influenced by renal capillary wedge pressure as well as by several extra-renal factors, such as heart rate, aortic stiffness/atherosclerotic burden and blood pressure. So, the RI has to be regarded in a broad context (see later).

### 6.1 Acute allograft rejection

B-imaging signs are swollen cortex with increased echogenicity (probably due to cellular infiltration) and prominence of the medullary pyramids. In mild/moderate rejection allografts can appear normal. Reduced perfusion can be an unspecific sign. The role of the resistive index is discussed later.

### 6.2 Acute tubular necrosis (ATN)

ATN is the most common cause of impaired renal function in the early posttransplant period (18). Ultrasonographic findings are nonspecific and include graft swelling, obscured corticomedullary differentiation and heterogenous areas of increased echogenicity as well as elevated RI (2).

### 6.3 Drug toxicity

Mostly calcineurin inhibitors (CNIs), e.g. tacrolimus, cause unspecific findings, such as graft swelling.

It is not possible to distinguish graft rejection from ATN and CNI toxicity *via* ultrasound, so in this case a biopsy has to be performed (8).

In the early period, drug induced acute interstitial nephritis can also be a reason for AKI in kidney allograft recipients

## 7 Other/late complications

As mentioned above, transplant artery stenosis is mostly a late complication, occurring until two years after transplantation.

Other late complications include chronic rejection, chronic nephrotoxicity of immunosuppressive drugs, nephrolithiasis and tumors. Especially for neoplasms, CEUS is an important diagnostic tool.

In posttransplant follow-up renal sonography screening is useful not only for the graft but also for the native kidney (urothelial malignancy, hydronephrosis,…).

## 8 Role of the renal resistive index (RRI)

The resistive index represents a marker of vascular impedance (28, 29) and is measured by Doppler spectrum analysis at the level of interlobar in the upper, mid and lower kidney pole.

RRI derives from the formula: (peak systolic velocity - end diastolic velocity)/peak systolic velocity, which expresses the percentage reduction of end-diastolic blood flow in renal vessels in relation to the maximum systolic blood flow (30). Thus, RI should be able to visualize the renal microcirculation.

Peak systolic velocity is determined by pulse pressure, left ventricular outflow and the distensibility of the late arteries and the aorta. Modifying pathological conditions are heart failure, aortic valve stenosis, aortic coarctation, aortosclerosis, and renal artery stenosis.

End diastolic velocity is determined by the heart rate, the renal capillary wedge pressure and the peripheral resistance. Modifying pathological conditions are heart failure, chronic or acute nephropathy, arteriolosclerosis, tachycardia, and drugs (betablockers, diuretics) (31).

RI is considered to be normal if it is < 0.7, indeterminate between 0.7 and 0.8, and elevated if > 0.8 (30). An elevated RI occurs in reduced or absent end-diastolic flow and is usually due to interstitial edema (32).

Hence, the RRI analysis is influenced by renal parameters as well as by systemic parameters and has to be interpreted in conjunction with the hemodynamic status (33). For example in heart failure there is systemic venous congestion, including renal venous pressure, due to the decreased cardiac output, leading to higher RRI indices (31, 34). To correlate these factors with the intravascular volume status of the patient, cavography may be used.

The role of RI measurement to predict graft outcome in kidney transplants is still controversial (35, 36):

A study by Naesens et al. showed that the routine measurement of the resistive index at predefined times after transplantation reflects mainly recipient factors (age, central hemodynamic factors) but not intrinsic characteristics of the allograft. At protocol-specified biopsy time points, changes in the RRI did not reflect changes in histologic features, whereas an increased RRI was shown, when biopsies were performed because of graft dysfunction. The previously described association of a resistive index of 0.80 or higher with graft survival could not be validated in this study (28). Accordingly, Heine et al. showed a poor correlation between allograft RRI and donor parameters such as age or allograft function, while graft RRI was strictly connected with host-related factors such as pulse pressure, IMT (intima-media thickness) and ABI (ankle brachial index) (37).

Jimenez et al. describe that an elevated RI (>0.9) in the postoperative period can be found in several types of graft dysfunction such as acute rejection, calcineurin inhibitor toxicity, severe acute tubular necrosis, renal vein obstruction, ureteral obstruction, and pyelonephritis. So, periodic RI measurement in the early postoperative period can be useful (38). In chronic allograft outcome, the RI was no surrogate marker of chronic graft pathology in protocol biopsies (39).

Kramann et al. reported that the RI obtained during the first 6 months after transplantation failed to predict renal allograft failure or death, whereas the RI measured 12–18 months after transplantation appeared useful to predict long-term allograft outcomes. In this study, patients with an RI >0.75 were 6-fold more likely to experience allograft failure or death than patients with a lower RI (40).

In a meta-analysis of Bellos et al. (2019) the role of RRI measurement in the post-transplant period and its efficacy in the prediction of delayed graft function was evaluated. In this study, high RI values were significantly associated with higher incidence of delayed graft function, pointing to the importance of RI measurement after kidney transplantation. This association was shown especially within the early postoperative period (5 days). Still, the diagnostic accuracy was described as moderate with a sensitivity of 52.5% and a specificity of 71.9%. The authors concluded, that - in order to the low sensitivity - RI measurement alone will not be adequately able to predict the occurrence of delayed graft function (27).

Another meta-analysis by Cano et al. showed that serial RI measurement in the early period after kidney transplantation is a valuable marker for determining renal graft function (41).

Loock et al. showed that a change in RRI ≥10% (ΔRI) between 4 months and 1 year after transplantation is a strong predictor of graft loss, whereas the absolute RI at both time points failed to be a significant predictor (RI cut-off ≥0.68) (42).

## 9 Discussion

To summarize, RI measurement seems to be an important predictor of renal graft dysfunction in the early postoperative period. In long-term renal allograft follow-up, its role remains uncertain. *Via* RI measurement, it is difficult to distinguish the relative contribution of renal histologic patterns (acute rejection, calcineurin inhibitor toxicity, acute tubular necrosis) and extrarenal central hemodynamic factors (arterial stiffness, leftventricular hypertrophy, age of the recipient).

We believe that an intra-individual change of RRI at any time is a warning sign and needs further examination. We also postulate that additional CCDS parameters like peak vascularization (POV) and sparing of peripheral vascularization (PVD) should be taken into consideration. Therefore, regular performed ultrasound examination plays a crucial role in the post-transplant follow-up care. Even in uncomplicated transplant history, sonography should be performed at least once a year to gain basic information about the transplanted organ. Whenever there is an acute problem, comparative images are available.

In any transplant sonography, the hydration status should be checked by cavography and hemodynamic parameters should be measured (at least the blood pressure). This is helpful for the interpretation of the RI. In our opinion, the RRI should be analyzed in combination with the overall perfusion of the organ (POV) and it can only be valued, if the peak systolic velocity is > 20cm/sec.

## 10 Summary and recommendation

The important role of ultrasound and especially CCDS in kidney transplant recipients is without controversy. This simple method is easily available and can be performed quickly with low cost. It is a powerful screening tool to detect acute and chronic causes of renal graft dysfunction. Especially in early diagnosis and management of structural and vascular complications, which may need surgical intervention to save the graft, it plays a crucial role.

Even though there are many controversies about the value and the prognostic relevance of the RRI, in our opinion the intraindividual progression is relevant, but only if interpreted in context with the actual hemodynamic and hydration status.

In conclusion, we believe that protocol sonographic survey of transplant patients as well as ultrasound in any acute graft dysfunction setting is necessary and irreplaceable.

## Author contributions

JS is trainee in sonography and internal medicine and wrote the draft of the manuscript as well as prepared the pictures. MT coauthored the manuscript, focused the discussion on the open issues and tried to summarize the experience of 20 years in transplant sonography for this introductionary mini review. All authors contributed to the article and approved the submitted version.

## Conflict of interest

The authors declare that the research was conducted in the absence of any commercial or financial relationships that could be construed as a potential conflict of interest.

## Publisher’s note

All claims expressed in this article are solely those of the authors and do not necessarily represent those of their affiliated organizations, or those of the publisher, the editors and the reviewers. Any product that may be evaluated in this article, or claim that may be made by its manufacturer, is not guaranteed or endorsed by the publisher.

## Abbreviations

ATN, acute tubular necrosis; AVF, arteriovenous fistula; CCDS, color coded duplex sonography; CEUS, contrast-enhanced ultrasound; CNI, calcineurin inhibitor; PA, pseudoaneurysm; POV, percentage of vascularization; PSV, peak systolic velocity; PVD, periphery vessel distance; RRI, renal resistive index; RVT, renal vein thrombosis; TRAS, transplant renal artery stenosis; US, ultrasound.
